# Knowledge, Attitudes, and Practices Among Nurses in Pakistan Towards Diabetic Foot

**DOI:** 10.7759/cureus.3001

**Published:** 2018-07-19

**Authors:** Muhammad Bilal, Abdul Haseeb, Abdur Rehman, Mohammad Hussham Arshad, Aashir Aslam, Sana Godil, Mohammad A Qamar, Saif N Husain, Muhammad H Polani, Araib Ayaz, Altamash S Ghazanfar, Zaki M Ghazali, Khurram A Khoja, Maarij Malik, Hania Ahmad

**Affiliations:** 1 Medicine, Dow University of Health Sciences (DUHS), Karachi, PAK; 2 Medicine, The Wright Center for Graduate Medical Education, Scranton, USA; 3 Medicine, Aga Khan University, Karachi, PAK; 4 Medicine, Karachi Grammar School, Karachi, PAK; 5 Medicine, Karachi Grammar School, Karachi , PAK; 6 Medicine, St. Michael’s Convent School, Karachi, PAK; 7 Medicine, The Lyceum School, Karachi , PAK

**Keywords:** diabetic foot ulcers, macdonald’s standard learning outcome, evidence based practice

## Abstract

Introduction

Diabetic foot ulcers are a pressing complication of diabetes mellitus. Wound care requires a significant proportion of healthcare resources. It is imperative, therefore, for healthcare professionals to possess sound knowledge of the disease along with a positive attitude to ensure better clinical practice. Our literature search revealed a scarcity of data pertaining to diabetic foot ulcers. Therefore, this study aims to evaluate the knowledge and attitudes of nurses regarding diabetic foot care.

Methods

A cross-sectional study design was employed, a pre-validated and pre-tested questionnaire was used to collect data from a sample size of 250 nurses working at two tertiary care hospitals in Karachi, Pakistan. The study was conducted over a period of three months (January to March 2018) and included all nurses who possessed at least one year of clinical experience in diabetic ulcer care. The statistical software employed was SPSS version 19 (IBM Corp., Armonk, NY, US). Non-parametric tests and descriptive statistics were used for data analysis and statistical significance was assumed at a p-value of less than 0.5.

Results

Only 54% of the nurses in our study possessed adequate knowledge of diabetic foot ulcers. The mean score of knowledge was 74.9 (±9.5). Macdonald’s standard criteria for learning outcomes was used to gauge the knowledge levels of our study population. Nurses performed best in the domain of ulcer care with 65.3% of the participants possessing good knowledge of the topic. The overall attitude of nurses towards patients with diabetic ulcers was positive.

Conclusion

This study highlights important gaps in nurses’ knowledge and sheds light on the lack of evidence-based practice. Poor knowledge can compromise healthcare standards, even with the presence of positive attitudes. Hence, a comprehensive revision of nursing curricula across local tertiary hospitals for allowing nurses to update their knowledge is warranted.

## Introduction

Diabetes mellitus (DM) is an alarming public health issue affecting one in every 11 adults (425 million cases worldwide). In Pakistan, approximately 7.5 million people suffer from the disease [1]. Diabetes mellitus usually presents with a multitude of chronic complications often associated with hyperglycemia, which include, amongst others, neuropathy, coronary artery disease, cerebrovascular disease, and peripheral vascular disease [2]. Approximately 10% of the patients have either one or both of peripheral neuropathy (PN) and peripheral vascular disease (PVD) when they are diagnosed with diabetes mellitus [3]. The aforementioned diseases are risk factors for foot disease. Diabetic foot refers to an area of necrosis or gangrene distal to the ankle in a diabetic patient [4]. An increased prevalence of diabetes is associated with a corresponding increase in diabetic foot disease. The global prevalence of a diabetic foot ulcer (DFU) is 3%-10% whereas there is a 15 % chance of diabetics developing a DFU during their lifetime [3].

DFUs are a common cause of hospitalization amongst patients with DM and are susceptible to frequent infections, which ultimately necessitates amputations of lower extremities. The disease inflicts a socioeconomic burden and is found to markedly decrease the quality of life among patients [5]. It is imperative for patients and healthcare professionals alike to have a comprehensive knowledge of foot care to prevent diabetic foot complications. Nurses should be able to impart quality care to patients and be effectively able to control the progression of the disease. The preventative strategy offered by nurses should ideally initiate from the identification of the patient and clinical examinations. This should be followed by briefing the patient with risk guidelines and appropriate care procedures [6]. Emphasis should be placed on the role of nurses in a health team, and they should be provided access to comprehensive education on wound management and care [7]. The construction of any sound educational program would require an evaluation of the current level of awareness about DFU among nurses in the healthcare setup.

There have been multiple studies that focus on knowledge of foot care amongst diabetics in Pakistan [8]. However, there is a paucity of data about wound-care practices among healthcare professionals in general and nurses in particular. Hence, the primary objective of our study is to evaluate the knowledge, attitudes, and practices of nurses towards the diabetic foot, at the tertiary care hospitals in Karachi.

## Materials and methods

This is a cross-sectional descriptive study conducted at two tertiary care hospitals (Dr. Ruth Km Pfau Civil Hospital and Jinnah Hospital) in Karachi. The study was conducted from January to March 2018. It was approved by the Institutional Review Board of Dow University of Health Sciences (DUHS) with approval number DUHS-11697. A sample size of 250 was selected for the study and verbal informed consent was obtained from all the participants. A 100% response rate was observed.

The inclusion criteria entailed that all nurses working in surgical wards and their outpatient departments and are directly involved in diabetic ulcer care should be included in the study. Nurses possessing professional experience would be better able to provide more-important information related to diabetic foot care, as compared to inexperienced nurses. Additionally, it was made sure that the nurses had at least one year of relevant clinical experience.

Non-probability convenience sampling was used and a pre-structured questionnaire was used to assess the knowledge, attitudes, and practices of nurses pertaining to diabetic foot care. The questionnaire was made bilingual (English and Urdu - the common language spoken by the general populace) to increase comprehension. The validity of the study tool was assessed by faculty experts reviewing its contents and a Crohn Bach’s alpha test was conducted, which returned 0.73. Hence, the study tool was considered acceptable for use. Additionally, a pilot study was conducted on 30 nurses to adapt the questionnaire to the reality of subjects. The results of the pilot study were excluded from the final set of results. Interviewer bias was removed by imparting instructions to interviewers on how to respond to the general queries of the interviewees. The questionnaires didn’t ask for the names of the participants and, hence, maintained confidentiality throughout the process.

The questionnaire, based on three sections, was derived from a similar study conducted on nurses working at teaching hospitals in Sri Lanka [9]. The personal and occupational data of nurses were obtained mainly through the first section of the questionnaire. It included items such as age, gender, professional experience, and qualifications.

The purpose of the second section of the study tool was to gauge the level of knowledge possessed by nurses about diabetic foot ulcers and their management. The multiple-choice format has greater validity over its open-ended counterpart. Hence, the knowledge section comprised 15 items focusing on risk factors, descriptions, and the management of diabetic ulcers. The three options provided for each question were “Yes,” “No,” and “I do not know,” The scoring criteria worked as follows: A score of 1 for each correct answer whereas incorrect answers and questions marked “I do not know” received a score of zero. A mean score was generated using the results and the level of knowledge classified into two groups. Nurses who scored equal to or greater than the mean score for knowledge were presumed to have good knowledge about diabetic foot care. In contrast, poor knowledge was classified as a score lesser than the mean value for knowledge.

The attitudes of nurses in relation to diabetic foot care were explored in the third section of the study instrument. This portion comprised 10 items, which were derived from the study conducted by Kumarasinghe et al. [9]. The basic themes covered in this section centered around the risk perception of diabetic foot ulcers, their clinical priority, and the professional interest shown by nurses towards diabetic foot care. A shift to the 5-point Likert scale was opted for this section. An assessment of attitudes on a wider scale is facilitated more by the Likert system as compared to the multiple-choice format offering dichotomous responses.

Data analysis

Once data collection was complete, the data were entered in SPSS version 19 (IBM Corp. Armonk, NY, US) for analysis. The computation of frequencies, percentages, and mean and median scores was conducted through descriptive statistics to evaluate knowledge and attitude scores. The data followed a skewed pattern and, hence, the Mann Whitney U test and Kruskal-Wallis test were employed for the analysis. All the categorical variables in our data had two possible responses except for “wound care interest.” Hence, we used the Kruskal-Wallis test to determine the association between the scores on nurses’ knowledge and the aforementioned variable. The relation between the knowledge and attitude scores was evaluated through Spearman’s coefficient. For all these statistical tests, the level of significance was set at a p-value of less than 0.05.

## Results

The percentage of female participants in the study was staggeringly high as compared to their male counterparts and accounted for 91.2% of the study population. The highest percentage of correct responses to a question (98%) were regarding the necessity to clean infected wounds. The demographic characteristics of the nurses are shown in Table 1. Most of the nurses were employed in surgical intensive care units (84.8%). Of the nurses interviewed, 47.2 % were less than or equal to 30 years of age.

**Table 1:** Demographic Features of the Study Subjects

Variable	N	%
Sex		
Female	228	91.2
Male	22	8.8
Age		
≤30	118	47.2
31–40	73	29.2
41–50	43	17.2
51–60	16	6.4
Professional qualification		
Diploma	205	82.0
Post-basic diploma	10	4.0
Degree	35	14.0
Nursing experience (in years)		
≤5	100	40.0
6–10	58	23.2
11–15	28	11.2
16–20	21	8.4
>20	43	17.2
Wound care experience (in years)		
≤5	133	53.2
6–10	50	20.0
11–15	35	14.0
16–20	18	7.2
>20	14	5.6
Formal training in wound care		
Yes	18	7.2
No	232	92.8
Current professional development activities		
No	168	67.2
In-service education	28	11.2
In a degree programme	35	14.0
Other	19	7.6

The mean score for nurses’ knowledge was 74.9 (±9.5). An evaluation of the nurses’ knowledge revealed that 13.0% and 15.0% of the nurses had very low or low knowledge, respectively. Nineteen percent of the nurses had a moderate level of knowledge whereas 40% of the study sample possessed high knowledge. Only 14% of the participants had very high levels of knowledge regarding diabetic ulcer care. The nurses’ knowledge on individual questions is shown in Table 2. The four criteria to assess knowledge and their respective mean scores were as follows: predisposing factors = 75 (±21.0), characteristics of ulcers = 76.0 (±24.3), complications of ulcers = 78.5 (±22.6), and ulcer care = 80.7 (±17.3). The two domains in which a higher percentage of nurses possessed good knowledge were predisposing factors and ulcer care (55% and 65.3%, respectively). A majority of the nurses fared poorly in their knowledge of the characteristics and complications of ulcers with only approximately 40% of the study population having a good score in the two domains.

**Table 2:** Frequency and Percentage Distribution of Nurses’ Knowledge on Diabetic Ulcer Disease

Item	Correct %	Incorrect %	Don’t Know %
Neuropathy is the predominant factor responsible for diabetic ulcers (True)	58.0	29.0	13.0
Sensory neuropathy results in unnoticed skin damages, which lead to the formation of ulcers (True)	96.0	2.5	1.5
Autonomic neuropathy is associated with dry skin, which predisposes to ulcer formation (True)	87.0	11.0	2.0
Diabetic neuropathic ulcers are typically found on weight-bearing areas of the foot (True)	68.0	24.0	8.0
Diabetic ischemic ulcers are less painful than diabetic neuropathic ulcers (False)	70.0	23.0	7.0
Neuropathy can be excluded if the foot skin is cool and pulses are absent (False)	79.0	11.0	10.0
The risk of amputation is higher when diabetic foot ulcer is associated with limb ischemia (True)	45.0	50.0	5.0
Presence of slough is not an indication of infection in diabetic ulcers (False)	94.0	2.0	4.0
Presence of osteomyelitis impairs the healing of diabetic ulcers (True)	86.0	6.5	7.5.0
Wound healing progress is unsatisfactory if the wound bed appears pink (False)	89.0	9.5	1.5.0
Mechanical offloading should be advised to facilitate ulcer healing (True)	42.0	48.0	10.0
Hyperbaric oxygen therapy is recommended for ulcer healing even in a well-perfused foot (False)	96.0	3.5	0.5
Infected, highly exuding wounds should be cleansed daily (True)	97.0	0.0	3.0
Iodine dressings are effective for wounds with clinical signs of infection (True)	68.0	24.0	8.0
Hydrogel dressings are useful to rehydrate the wound bed and control the moisture in wounds (True)	86.0	9.5	4.5

Table 3 depicts the association between knowledge and demographic features. There was a significant association between nurses’ knowledge and their nursing (0.006) and wound care experiences (0.007). It is interesting to note that only the time period of one to five years was significant for both domains. Significant associations were also observed between nurses’ knowledge, receiving wound care training (0.002), and working in outpatient departments (0.021). However, we observed no statistically significant associations between knowledge and gender, age and professional qualifications. A substantial proportion of the nurses identified their knowledge as satisfactory (70.5%) whereas only 0.5% of them thought their knowledge was poor. Of these, 27.0% believed they possessed a good knowledge of diabetic ulcer disease. Only 2% of nurses, on the other hand, rated their knowledge as excellent.

**Table 3:** Factors Associated with Nurses’ Knowledge OPD: outpatients department

Characteristic	N	Median	IQR	p-value
Sex				
Female	228	78.0	15.1	0.371
Male	22	75.3	25.6	
Age				
≤30 years	118	78	19.0	0.071
>30 years	132	78	14.4	
Professional qualification				
Diploma	205	78	14.4	0.342
Degree	245	72	25.1	
Nursing experience				
1–5 years	100	71.0	19.0	0.006*
> 5 years	150	85.0	14.3	
Wound care experience				
1–5 years	133	71.0	19.0	0.007*
>5 years	117	85.0	14.3	
Wound care training				
Yes	18	88.0	19.0	0.002*
No	232	71.0	19.0	
Hospital unit				
OPD	212	71.0	18.0	0.021*
Surgical Wards	38	85.0	12.3	

Educational activities (70.2%) and knowledge sharing with peers (80.9%) were by far the most popular methods utilized by our study population to improve their knowledge. Scientific journals (65.2%) and books (55%) were other common methods used by nurses. Only 30% of the nurses though were dependent on the Internet as a knowledge source. Nevertheless, there was no significant association between overall knowledge and any knowledge-updating sources.

The highest and lowest possible scores on the attitude scale were 10 and 50, respectively. Our study, however, manifested a range of attitude scores from 12 to 48. An analysis of the median score (38, range 21-48) for the study revealed that the overall attitude of nurses towards patients with diabetic ulcers was positive. Table 4 shows a detailed assessment of individual attitudes and highlights that a majority of the nurses prioritized ulcer prevention over treatment (85.6%), gave diabetic ulcer care a high clinical priority (92.8%), and considered it their responsibility to advise patients on avoiding re-ulceration (87.6%). There was a significant difference observed between the nurses’ attitudes and their age (p=0.022). Nurses who were younger than 40 years of age had more positive attitudes (median = 44.00) than older nurses (median = 39.00). Nevertheless, there was no statistical significance observed between nurses’ attitudes and their gender and wound care experience. Additionally, Spearman’s coefficient between nurses’ knowledge and attitudes was calculated to be q=0.121. Hence, there was no statistical correlation established. Ninety-eight percent of the nurses demonstrated an interest in diabetic ulcer care whereas 70% of the study cohort expressed their desire to pursue a training course in ulcer care. However, only a very small proportion of the study sample (5%) wanted to undertake research in ulcer care. Furthermore, there was a statistically significant association between nurses’ interest in ulcer care and their knowledge (p = .031*) and attitudes (p = .0003*). It was generally observed that nurses with a good knowledge and positive attitudes were more inclined to participate in training courses and research projects regarding ulcer care.

**Table 4:** Nurses’ Attitudes to Diabetic Ulcer Care

Item	Strongly Agree n (%)	Agree n (%)	Neither agree nor disagree n (%)	Disagree n (%)	Strongly disagree n (%)
1. I think diabetic ulcer treatment is more important than ulcer prevention	13 (5.2)	8 (3.2 )	15 (6.0 )	125 (50.0 )	89 (35.6 )
2. I do not think it is necessary to assess diabetic ulcers regularly	5 (2.0 )	5 (2.0 )	8 (3.2)	138 (55.2 )	94 (37.6 )
3. Diabetic ulcer care is too time-consuming for me to carry out	3 (1.2 )	25(10.0)	40 (16.0 )	125 (50.0 )	57 (22.8 )
4. In comparison with other areas of nursing care, diabetic ulcer care is a low priority task for me	3 (1.2 )	8 (3.2 )	25 (10.0 )	120 (48.0 )	94 (37.6 )
5. If I have the opportunity, I would like to avoid caring for diabetic ulcers	5 (2.0 )	3 (1.2 )	10 (4.0 )	58 (23.2 )	174 (69.6 )
6. I do not have time to advise each patient individually on how to look after their ulcers	5 (2.0 )	18 (7.2 )	30 (12.0 )	118 (47.2 )	79 (31.6 )
7. It is not my responsibility to educate patients with diabetic ulcers on how to reduce re-ulceration	0 (0.0 )	13(5.2)	18 (7.2 )	130 (52.0 )	89 (35.6 )
8. I cannot think about pain when cleaning diabetic ulcers	5 (2.0 )	88 (35.2 )	30 (12.0 )	103 (41.2 )	24 (9.6 )
9. I do not like to care for diabetic ulcers in my practice	0 (0.0 )	25 (0.0 )	30 (12.0 )	150 (50.0 )	45 (18.0 )
10. I do not get satisfaction by caring for diabetic ulcers	0 (0.0 )	0 (0.0 )	15 (6.0 )	137 (54.8 )	98 (39.2 )

## Discussion

An extensive literature search on diabetic foot ulcers revealed that there is a paucity of local and global data focusing on knowledge of healthcare workers and their attitudes. A study conducted in a Sri Lankan clinical setting coincides with our observation. Our data revealed a large gender gap, with females constituting a predominant portion of the interviewed workforce. This pattern of gender disparity has been observed in multiple nursing studies worldwide [10]. Furthermore, approximately 53% of the nurses were older than 30 years of age. An aging workforce, while more experienced, is also more prone to develop various disorders due to intense workload [11]. Our data indicate that although 46.8% of the nurses possessed wound care experience of more than 5 years, not even 1% of the sample population had received formal wound care training. A similar finding was reported by a Swedish study where nurses lacked comprehensive wound care training despite a decade of professional experience [12]. A study in Ethiopia revealed that 91.1% of participants lacked any wound care training [10]. This is a worrisome finding because a lack of training can serve as a potential barrier for nurses to translate their preexisting knowledge on ulcer care into practice [13]. In accordance with the Macdonald’s standard of learning outcomes, only 54% of the participants were adequately knowledgeable (range 80-100) [14]. This finding is in line with a study conducted in Sri Lanka, which reported similar knowledge scores [10].

Additionally, a survey in Bangladesh revealed that the mean knowledge of nurses concerning the prevention of diabetic foot ulcers was only 52.60% [15]. The current level of knowledge revealed by our study is unsatisfactory because the nurses are employed in tertiary care hospitals and are expected to possess comprehensive knowledge [10]. A low level of knowledge can be attributed to the participants’ basic knowledge and their professional expertise. Only 14.0% of the nurses had a basic degree whereas 53.2% of the participants had wound care experience of less than or equal to five years. It can also be argued that basic nursing degrees and diplomas are not centered around updated information pertaining to ulcer care [13]. An individual analysis of the four knowledge domains revealed that the highest percentage of nurses possessed a good knowledge of ulcer care. However, they depicted a poor grasp of the characteristics and the complications of ulcers. These individual knowledge findings can reflect the primary focus of nursing curricula at tertiary care hospitals.

The assessment of knowledge on individual items helps assess the primary features of the nurses’ routine practices. Ninety-four percent of the nurses were aware that slough presence is indicative of infection in diabetic ulcers. Nevertheless, routine practices have been found to influence nurses’ clinical acumen and are not commonly updated. This redundancy in clinical practice can be attributed to the general lack of knowledge in the nursing workforce [16]. A question aimed at evaluating risk assessment for amputations in DFUs was answered incorrectly by 50% of the nurses. A poor knowledge of risk assessment was also observed in a Nigerian study where 73% of participants had incorrect responses [13]. This is in contrast to a multicenter study conducted in Sweden, which reported high scores for risk assessments in pressure ulcers [12]. It is eminent that nurses specialize in evidence-based practice to comprehensively prevent and manage diabetic foot ulcers. Forty-eight percent of the nurses were unaware of the significance of mechanical offloading for the healing of DFUs. This reflects that evidence-based practice has been ignored in the nursing curriculum. The survey on nurses’ knowledge in Bangladesh also concluded that their clinical settings did not primarily focus on evidence-based care [15].

Our study found a significant association between nurses’ knowledge and their wound care and nursing experiences, wound care training, and work units (surgical intensive care units (ICUs) and outpatient departments (OPDs)). Surprisingly, our study found that nurses’ knowledge was significantly correlated with a professional and wound care experience of five years or less. This finding coincides with a study conducted at Brazilian University Hospital, which revealed that nurses with greater years of experience had lower knowledge scores [17]. A study at the University of Copenhagen found no statistical link between nurses’ knowledge and the duration of their professional experiences [18]. However, there have been various studies that report contrasting findings and establish a significant association between nurses’ knowledge and years of experience [13]. This finding of the study can help us to establish a dual conclusion. It possibly reflects that younger graduates have a more comprehensive knowledge of nursing principles. Furthermore, this also demonstrates a lack of effort put in by older nurses to update their skills [16]. We also observed that nurses in OPDs had better knowledge, as compared to those working in surgical ICUs. This can possibly be linked to a higher frequency of patients in outpatient departments and, hence, providing for greater clinical exposure to nurses. However, contrasting findings have been reported in other studies where nurses deployed in surgical units performed better on knowledge tests [19]. Multiple studies have reported a significant link between wound care training and the knowledge possessed by nurses [10]. An Ethiopian study on pressure ulcer prevention observed higher levels of knowledge in nurses who had received formal wound care training [10]. A possible explanation could be greater clinical exposure acquired through training, which facilitates learning. Our study found no statistical significance between nurses’ knowledge and gender, professional qualifications, and age.

Various nursing studies have explored nurses’ self-perception of knowledge and the common sources used by them to enhance their knowledge. In our study, 70.5 % of the nurses identified their knowledge as satisfactory. This finding coincides with a similar study on Sri Lankan nurses [10]. A potential explanation could be that nurses might not be aware of the limitations in their current knowledge, which may have created a false sense of confidence [19]. Knowledge sharing with colleagues and various educational activities were the most popular knowledge-enhancing sources used by participants in the study. A study conducted in Huddersfield, UK, found that a majority of nurses are dependent on professional development programs for improvements in knowledge [19]. An inclination towards these methods could, however, be a consequence of lack of access to technology at the workplace. An important limitation of obtaining knowledge from colleagues could be ignorance of ideal practice methods because specialists can generally mold guidelines to suit their time constraints [20].

A multitude of studies have observed positive attitudes among nurses towards ulcer care and the data in our study brings us to the same conclusion [10]. It has been established that attitude is an influential factor in determining the intention of an individual [16]. A positive attitude, for instance, can markedly increase preventative measures for a disease [21]. Nevertheless, it is important to recognize contrasting results. A clinical study conducted in Saudi Arabia documented unsatisfactory scores for nurses’ attitude and reported that 10.7 % of the participants believed that prevention of ulcers is a time-consuming process [22]. Translation of attitudes into work practice can be hindered by various barriers. Moore and Price identify a shortage of time and work personnel as two important factors in limiting effective ulcer care [16]. A shortage of staff can push ulcer care and prevention down the priority list although our current results indicate that 85.4% nurses perceive DFU care as a high-priority task. Our study found that age significantly influenced nurses’ attitudes towards ulcer care with younger nurses harboring a more positive attitude. Nevertheless, there was no significant association between the nurses’ knowledge and attitudes. In contrast, a study conducted on Belgian nurses concluded a correlation between knowledge and attitudes and established a link between work practices and attitudes [23]. In our study, nurses’ attitudes were not influenced by their wound care experiences. This finding coincides with the results of the study conducted by Beeckman et al. where nurses’ attitudes were unaffected by formal wound care training [23]. This observation demonstrates the limited utility of current educational methods to influence workplace attitudes and, ultimately, practice. This also necessitates effective interventions to improve attitudes and clinical practices.

A majority of the nurses in our study demonstrated a great interest in diabetic ulcer care. Nurses’ interest was found to significantly influence their attitudes and knowledge. Moreover, nurses with good knowledge and positive attitudes were more inclined to participate in ulcer training courses and research projects. Nevertheless, only 5% of nurses showed an interest in pursuing ulcer care research. The merits of research in advancing evidence-based practice should be further highlighted to increase participation among nurses.

## Conclusions

This study reflects that nurses generally possess an inadequate level of knowledge pertaining to ulcer care despite a positive attitude. Formal wound care training and work experiences demonstrated a significant correlation with knowledge. A comprehensive educational program focusing on evidence-based practice is necessary to ensure positive attitudes and better clinical practices. Evidence-based clinical practice relies heavily on research. Nurses should be made aware of the importance of research in their clinical practice and be provided with opportunities to partake in research activities.
